# UL28 and UL33 homologs of Marek’s disease virus terminase complex involved in the regulation of cleavage and packaging of viral DNA are indispensable for replication in cultured cells

**DOI:** 10.1186/s13567-021-00901-5

**Published:** 2021-02-12

**Authors:** Aijun Sun, Shuaikang Yang, Jun Luo, Man Teng, Yijie Xu, Rui Wang, Xiaojing Zhu, Luping Zheng, Yanan Wu, Yongxiu Yao, Venugopal Nair, Gaiping Zhang, Guoqing Zhuang

**Affiliations:** 1 College of Veterinary Medicine, Henan Agricultural University, Zhengzhou, 450002 Henan China; 2 International Joint Research Center of National Animal Immunology, College of Veterinary Medicine, Henan Agricultural University, Zhengzhou, 450002 Henan People’s Republic of China; 3 Key Laboratory of Animal Immunology, Ministry of Agriculture and Rural Affairs & Henan Provincial Key Laboratory of Animal Immunology, Henan Academy of Agricultural Sciences, Zhengzhou, 450002 China; 4 UK-China Centre of Excellence for Research On Avian Diseases, Henan Academy of Agricultural Sciences, Zhengzhou, 450002 China; 5 The Pirbright Institute & UK-China Centre of Excellence for Research On Avian Diseases, Pirbright, Ash Road, Guildford, GU24 0NF Surrey UK

**Keywords:** Marek’s disease virus, DNA packaging, UL28, Co-transfection, UL33

## Abstract

Processing and packaging of herpesvirus genomic DNA is regulated by a packaging-associated terminase complex comprising of viral proteins pUL15, pUL28 and pUL33. Marek’s disease virus (MDV) homologs UL28 and UL33 showed conserved functional features with high sequence identity with the corresponding Herpes simplex virus 1 (HSV-1) homologs. As part of the investigations into the role of the UL28 and UL33 homologs of oncogenic MDV for DNA packaging and replication in cultured cells, we generated MDV mutant clones deficient in UL28 or UL33 of full-length MDV genomes. Transfection of UL28- or UL33-deleted BAC DNA into chicken embryo fibroblast (CEF) did not result either in the production of visible virus plaques, or detectable single cell infection after passaging onto fresh CEF cells. However, typical MDV plaques were detectable in CEF transfected with the DNA of revertant mutants where the deleted genes were precisely reinserted. Moreover, the replication defect of the UL28-deficient mutant was completely restored when fragment encoding the full *UL28* gene was co-transfected into CEF cells. Viruses recovered from the revertant construct, as well as by the *UL28* co-transfection, showed replication ability comparable with parental virus. Furthermore, the transmission electron microscopy study indicated that immature capsids were assembled without the UL28 expression, but with the loss of infectivity. Importantly, predicted three-dimensional structures of UL28 between MDV and HSV-1 suggests conserved function in virus replication. For the first time, these results revealed that both UL28 and UL33 are essential for MDV replication through regulating DNA cleavage and packaging.

## Introduction

Herpesviruses are categorized to α, β and γ subfamilies based on genome structure and biology [1]. Depicted by the cryo-EM structure, mature herpesvirus particle contains the well-organized double-stranded DNA (dsDNA), an icosahedral capsid, a capsid-associated tegument layer wrapped by a lipid envelop [2]. Marek’s Disease Virus (MDV), belonging to the *Alphaherpesvirinae* subfamily, includes the serotype 1 (MDV-1, *Gallid alphaherpesvirus 2*), serotype 2 (MDV-2, *Gallid alphaherpesvirus 3*) and serotype 3 (*Meleagrid alphaherpesvirus 1*, herpesvirus of turkeys, HVT). While serotypes 2 and 3 are non-pathogenic and used as vaccines, MDV-1 is pathogenic causing diverse degree of pathogenesis including immunosuppression, neurological diseases and tumors in their natural avian hosts depending on virulence of isolates [3, 4]. In vitro, highly cell-associated MDV replication induces characteristic plaques on infected cell monolayers, which could be used as an excellent cell model for virus replication analysis. Similar to other α-herpesvirus, the genome structure of MDV comprises of a unique long (UL) and unique short (US) region, flanked by terminal and inverted repeat long (TRL/IRL), and terminal and inverted repeat short (TRS/IRS) regions, respectively (Figure 2A). The approximately 180 kb MDV genome encodes more than hundred proteins, most of which are yet to be fully characterised [5, 6]. Genome alignment analysis revealed the high conservation of MDV serotypes especially in UL and US regions, which encodes highly conserved virus replication related elements, such as glycoproteins, tegument proteins and capsid-associated proteins [5, 6]. MDV encodes a specific set of glycoproteins including gB,gC, gD, gE, gH, gI,gK, gL and gM. It has been shown that gB, gE, gI, gM and UL49.5 are essential for the MDV spread in cultured cells [7, 8]. However, gC exhibited negative effect for MDV replication in vitro [9]. Meanwhile, MDV-encoded tegument proteins perform both regulatory and structural roles in the viral replication. For instance, one of the major tegument protein VP22, encoded by UL49, was indispensable for the propagation of MDV mainly relying on its N-terminus central domain, which might affect viral replication through modulating cell cycle metabolism [10]. The tegument proteins in cluster UL46–UL49 are conserved and play different roles in viral replication. For example, UL46 and UL48 have functions associated with capsid formation. While UL47 was found to be expressed at very low level in infected cultured cells, UL46, UL48 and UL49 showed enhanced expression suggesting a potential regulatory role in viral replication [11].

Although tremendous progress in understanding the molecular mechanisms involved in MDV pathogenesis has been achieved in the past decades, key determinants of MDV replication are still largely unknown. In particular, there is a large gap in our understanding of the MDV packaging mechanism that governs the viral assembly and maturation. The quality control steps of assembly and DNA packaging are critical to ensure herpesvirus replication regulated by multiple highly conserved viral proteins. DNA packaging is one of the critical determinants in viral maturation, in which monocatemeric form cleaved from replicated concatemeric dsDNA was translocated into preformed viral capsids by an adenosine triphosphate (ATP)-driven terminase complex [12]. Previous studies have revealed that three types of capsids A, B and C were formed depending on different forms of DNA packaging. For instance, the capsid C was enveloped when the cleaved viral monocatemeric was correctly packaged into the preformed capsid shell. Once the DNA packaging is deficient, capsid A or B became “dead-end” products since no viral monocatemeric form was translocated. Mature capsid C, but not immature capsid A and B, was associated with infectivity [13]. As the powerful motor, the terminase complex drives viral genomic DNA cleavage and delivery into the capsid, which includes three core components of UL15, UL28 and UL33 [14]. Recent report on the high-resolution structure of the herpesvirus hexameric terminase complex has provided detailed insights into the structural features necessary for sequential revolution of DNA translocation and concerted cleavage for efficient packaging of their genomes [15]. The roles of these terminase complex-associated proteins are less well studied in most of herpesviruses, including MDV.

**Figure 1:**
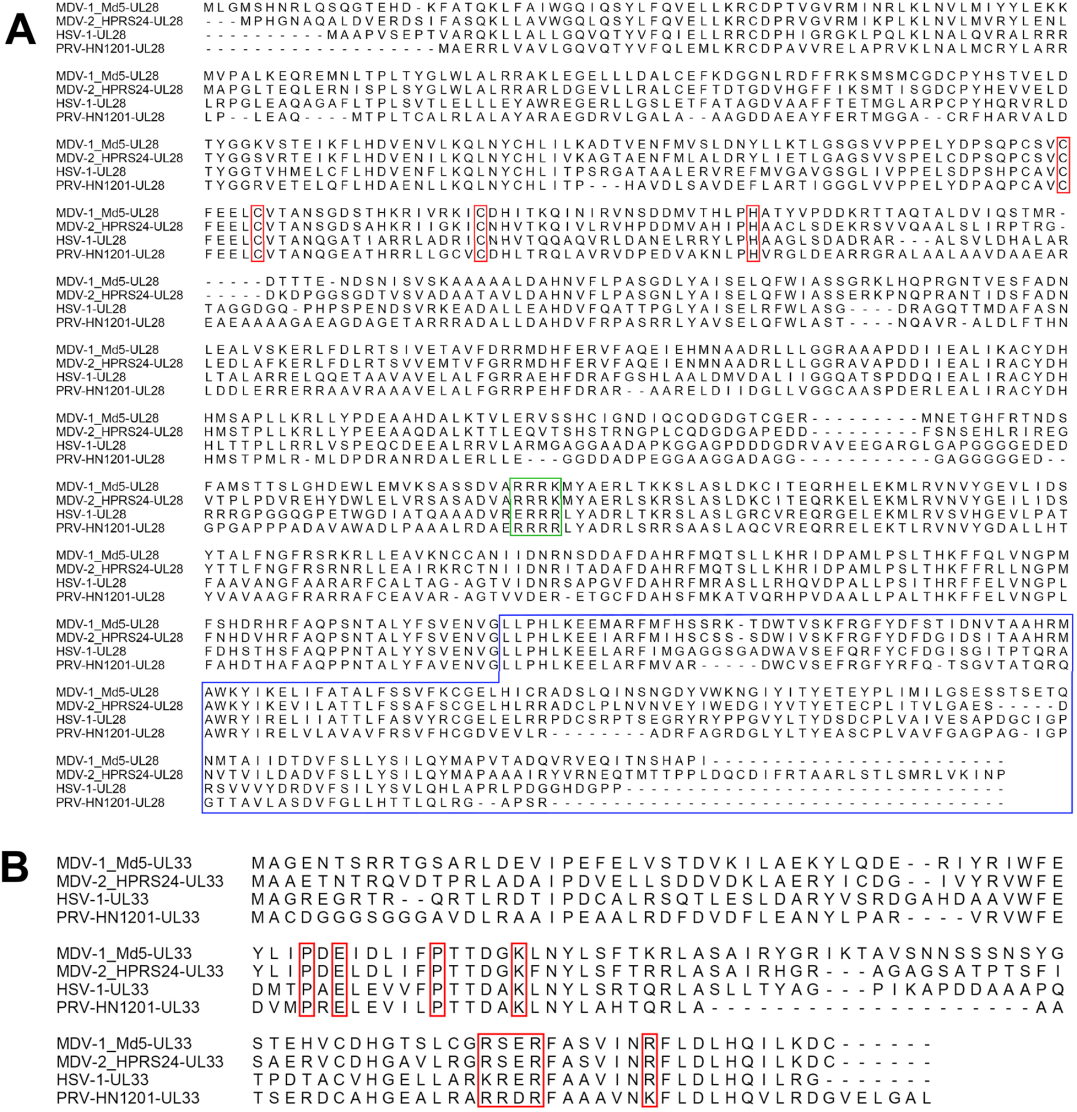
**Predicted highly conserved sequences of the UL28 and UL33 polypeptide chain in herpesviruses.** **A** The specific motifs of the UL28 were marked with red boxes, including potential nuclear localization sequence 462-RRQR-465, a putative C-X2-C-X22-C-X–H zinc-finger motif and a predicted conserved C terminal domain responsible for nuclear translocalization; **B** The highly conserved region of the UL33 marked with red boxes.

The MDV *UL15*, *UL28* and *UL33* genes located within the UL region are colinear with their counterparts of HSV-1, the prototypic α-herpesvirus [5, 6]. As important terminase components, UL28 and UL33 are highly conserved among the three MDV serotypes. Further homologous comparison analysis indicated moderate identity with other α-herpesviruses, implicating possible distinct roles in viral biological activities (Figure 1). However, the detailed role of the MDV UL28 and UL33, especially in its distinct cell-associated replication, has not been examined. Using mutagenesis approach, we demonstrate here that the UL28 and UL33 deficient MDV mutants were unable to form infectious virions. The revertant viruses with restored UL28 and UL33 expression were completely resumed in the replication ability. Moreover, when co-transfected with the purified PCR product encoding the entire *UL28* gene, the MDV ΔUL28 mutant was able to form virus plaques and grow in cultured cells. The revertant virus as well as the UL28 co-transfection restored viruses had comparable growth properties with parental virus. The role of the UL28 and UL33 in MDV genomic DNA packaging and replication was discussed.

## Materials and methods

### Cells, viruses, and reagents

Primary CEFs produced from specific pathogen free (SPF) chicken embryos were cultured in Dulbecco’s modified essential medium (DMEM) (Solarbio, China) containing 5% fetal bovine serum (FBS) (Gibco, USA). The recombinant viruses of MDV ΔUL28, MDV ΔUL33, MDV ΔUL28-Re and MDV ΔUL33-Re were generated from Md5BAC [16] MDV ΔUL28, MDV ΔUL33, MDV ΔUL28-Re and MDV ΔUL33-Re and *UL28*-cotransfected were all generated by calcium phosphate transfection into CEF monolayer cells with 500 ng BAC DNA. BAC DNA was isolated by plasmid MAXI kit (QIAGEN, Germany) according to the manufacturer’s instruction, other reagents which are analytic pure were purchased from China.

### PCR assay and qPCR assay

For PCR assay, genomic DNA was extracted from MDV uninfected or infected CEF cells using the phenol–chloroform method described previously [16]. The primers used in experiments were designed and synthesized by Shenggong (Sangon, Shanghai, China) (Table 1). The PCR assay was performed on a PowerCycler System (Analytik Jena AG, Germany). The PCR products were separated by electrophoresis on a 1% agarose gel and screened by Gel DocTM XR + Molecular Imager System (BIO-RAD, USA).

**Table 1:** The primers used in the experiments

Primers	Sequences	Purpose
Del UL28 kana F	CACACAAATCTTTATATTTTCTCACACGTCGGTTGTGCGGGATTGAAGCAGGATGACGACGATAAGTAGGG	Primers with the *UL28* homologous sequences for amplification of the *Kan*^*R*^ cassette gene
Del UL28 kana R	ATAAATTGTGTGAGATAAAATGCAGGGACACAAGAATATTGGACGGGTCAGCTTCAATCCCGCACAACCGACGTGTGAGAAAATATAAAGATTTGTGTGCAACCAATTAACCAATTCTGATTAG	
UL28 BamHI F	GATCGGATCCCAAATGGAGTTGGAGGAC	Primers for PCR identification of the *UL28* gene in Figure 3A
UL28 ApaI R	GATCGGGCCCGTTCTAGACGGATCACGG	
UL28 XHOI KANA.3	GATCCTCGAGCAACCAATTAACCAATTCTGATTAG	Primers for construction of the *UL28* revertant mutant
UL28 XHOI KANA.5	CTCGAGGCATTGGTATCCAAAGAACGTCTTTTCGATCTGAGAACCTCAATAGGATGACGACGATAAGTAGGG	
Meq Star US	CCGCACACTGATTCCTAG	Primers for PCR and RT-PCR amplification of the *meq* gene
Meq End US	CCTTTATGTTGATCTTCCCG	
Pet30a-UL28 BamHI F	GATCGGATCCTTGGGAATGTCTCATAACCG	Primers for PCR identification of the *UL28* gene in Figure 5A
Pet30a-UL28 NotI R	GATCGCGGCCGCAGATGGGGGCGTGGCTG	
UL28 F1	TTTCGATCTGAGAACCTCA	Primers for RT-PCR identification of the *UL28* gene in Figure 5B
UL28 R1	ATAATGCTGTATTCGACGG	
Del UL33 kana F	TTCTCCAGAAGATCGGATAAAAGCTGCCAACTCTGTAGGGCGGTTGGCCAGGATGACGACGATAAGTAGGG	Primers with the *UL33* homologous sequences for amplification of the *Kan*^*R*^ cassette gene
Del UL33 kana R	AACAGTCCTAACAGTCTTTCAAAATTTGATGTAAATCGAGGAACCTATTAGGCCAACCGCCCTACAGAGTTGGCAGCTTTTATCCGATCTTCTGGAGAACAACCAATTAACCAATTCTGATTAG	
UL33 HindIII F1	GATCAAGCTTATGCCACATAGGGTTC	Primers for PCR identification of the *UL33* gene in Figure 3B
UL33 BamHI R1	GATCGGATCCGTTCGGAATGACTTCCATC	
UL33 PstI Kana.5	GATCCTGCAGTGAGTAACAATAGCTCCTCAAATTCGTATGGAAGCACCGAGCACGTCTGAGGATGACGACGATAAGTAGGG	Primers for construction of the *UL33* revertant mutant
UL33 PstI Kana.3	GATCCTGCAGCAACCAATTAACCAATTCTGATTAG	

All qPCR assays were performed on a Quant Studio 5 Detection System (ThermoFisher, USA), and the results were analyzed by Quant Studio™ Design and Analysis Software Version 1.4.3. The 20 μL reaction contained 5 μL (10 ng/μL) of plasmid DNA or cellular genomic DNA, 1 μL of each primer (10 pmol), and 10 μL of FastStart Universal SYBR Green Master mix (ROX). The thermal cycling conditions were as follows: 40 cycles of 95 °C for 15 s followed by 60 °C for 1 min.

### Construction of mutant viruses by BAC mutagenesis

For construction of mutant viruses by BAC mutagenesis, a two-step lambda red-mediated homologous recombination was performed in *Escherichia coli* as previously described [17]. In brief, PCR using specific primers with homologous sequences was conducted with high fidelity DNA polymerase (ThermoFisher, Waltham, MA, USA). PCR product was purified and electroporated (1800 V, 100 Ω, 2.5 μF) into the BAC-containing competent cells. Then the competent cells were spread onto Luria–Bertani (LB) agar plate containing kanamycin (30 μg/mL), ampicillin (100 μg/mL) and chloramphenicol (30 μg/mL) until the bacterial colonies are observed. The triple antibiotic-resistant colonies were picked up and inoculated into fresh LB cultural media. In the recombination step, the final 0.2% arabinose was added to the growth medium resulting in induction of enzyme expression and cleavage.

### Characterization of growth properties in vitro

Briefly, CEFs seeded on 60-mm plates were inoculated with 100 plaque-forming units (PFUs) of each virus. On day 1, 3, and 5 post-inoculation, CEF cells were collected and MDV genome copy numbers were detected for virus titration. For MDV specific plaque area detection, the same amount of virus was inoculated into 6-well plate, and plaque areas were examined after 7 days post-infection.

### Indirect Immunofluorescence (IFA) Assay

CEF cells transfected with BAC mutants were washed with phosphate-buffered saline (PBS) and fixed with ice-cold acetone: methanol (6:4) at room temperature for 10 min. The cells were air-dried after discarding the fixation solution and subsequently blocked with 5% milk blocking solution for 2 h in room temperature. Cells were incubated with MDV gB-specific monoclonal antibody for 1 h at 37 °C. After washing off the antibody with PBS, cells were incubated for one hour with goat anti-mouse fluorescein isothiocyanate (FITC)-labeled secondary antibody. Cells were washed three times with PBS and examined using inverted fluorescence microscope (Olympus, Japan).

### Transmission electron microscopy

CEF cells were infected with the MDV or transfected with the MDV △UL28. Three days post-infection or transfection, the CEF cells were fixed on ice with 2.5% glutaraldehyde for 5 min, scraped and collected with a scraper, centrifuged at 1500 rpm for 15 min at 4 °C, replaced with the glutaric dialdehyde solution after 4 h of fixation. Cells were then fixed with 1% Osmic acid for 2 h, dehydrated with ethanol, polymerized with epoxy resin to make ultra-thin sections (LEICA EM UC7, Germany), stained for 20 min with saturated uranyl acetate aqueous solution, followed by treatment with lead citrate solution for 5 min, and then photographed by transmission electron microscope (JEM-1400, Japan).

### Prediction and establishment of the tertiary structure model of the UL28

Tertiary structure of both MDV-1 and HSV-1 encoded UL28 protein models were predicted by the I-TASSER server. The alignment and analysis of protein models were performed by Pymol 2.4.1 (Schrodinger, Palo Alto, CA, USA). The graphical presentation was prepared by Adobe illustrator 23.0 software (Adobe, CA, USA).

### Data and statistical analysis

The results of virus growth kinetics were analyzed by using the Graphpad Prism version 8.0.1 software (GraphPad Software, Inc. La Jolla, CA, USA). Two-way ANOVA statistical analysis was used for each data point, which represented an average of triplicates. A value of *P* < 0.05 was considered statistically significant.

## Results

### Predicted function of MDV UL28 and UL33

MDV UL28, with a 2,379-bp ORF, encodes a 793 aa protein with a predicted molecular mass of 90.1 kDa, while UL33, with a 402-bp ORF, encodes a 134 aa protein with a predicted molecular mass of 15.3 kDa. Alignment of the UL28 and UL33 sequences using the Clustal Omega software demonstrated highly conserved motifs. In UL28, 506-RRQR-509 motif conserved among many herpesviral families could act as a potential nuclear localization sequence [18]. Another highly conserved domain (C-X2-C-X22-C-X–H) within UL28 contains a putative zinc-finger motif, which is critical for the terminase function of DNA cleavage and packaging in other herpesviruses [19–21]. The conserved C terminus of MDV UL28 may be required for nuclear translocalization [19] (Figure 1A). Alignment of the UL33 showed several high conserved regions, which are critical for HSV-1 genomic DNA cleavage and packaging (Figure 1B) [22].

### Construction and identification of the *UL28* deleted and revertant mutants

**Figure 2:**
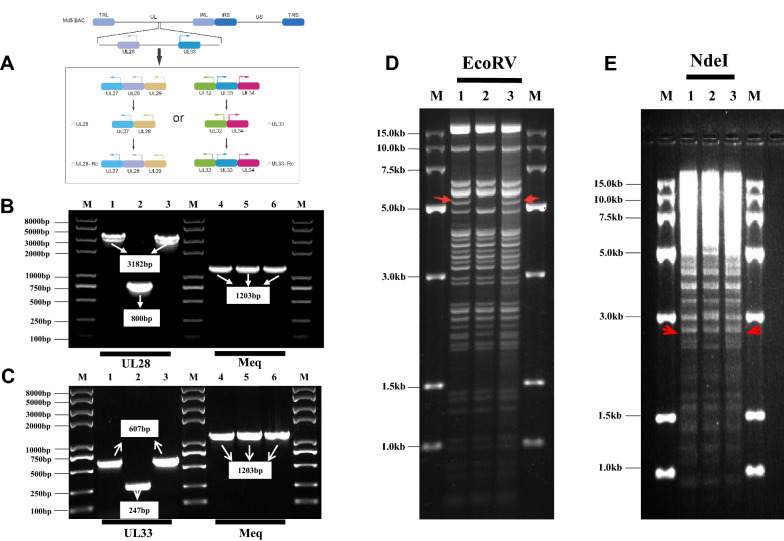
**Construction and Identification of the UL28 and UL33 gene deletion and revertant constructs.** **A** Schematic diagram represents a linear MDV genome consisting of a unique long (UL) region and a unique short (US) region. Both terminal repeats (TRL/TRS) and the internal repeat (IRL/IRS) regions locate on both sides of the UL and US region. Location of the *UL28* and *UL33* genes on MDV is also shown; **B** PCR analysis of the virus genome with *UL28* and *meq* specific primers. The lanes 1, 2 and 3 indicate the PCR amplification of the *UL28* gene using parental virus, MDV △UL28 or MDV △UL28-Re mutants as templates, respectively; The lanes 4, 5 and 6 indicate the PCR amplification of the *Meq* gene using parental virus, MDV △UL28 or MDV △UL28-Re mutants as templates, respectively; **C** PCR analysis of the virus genome with the *UL33* and *meq* specific primers. The lanes 1, 2 and 3 indicate the PCR amplification of the *UL28* gene using parental virus, MDV △UL33 or MDV △UL33-Re mutants as templates, respectively; the lanes 4, 5 and 6 indicate the PCR amplification of the *Meq* gene using parental virus, MDV △UL33 or MDV △UL33-Re mutants as templates, respectively; **D** RFLP analysis of the genomic DNAs of the parental virus, MDV △UL28 or MDV △UL28-Re. DNA was digested with EcoRV. lane 1 indicates the parental virus; lane 2 represents MDV △UL28; lane 3 indicates MDV △UL28-Re; **E** RFLP analysis of the genomic DNAs of the parental virus, MDV △UL33 or MDV △UL33-Re. DNA was digested with NdeI. lane 1 indicates the parental virus; lane 2 represents MDV △UL33; lane 3 indicates MDV △UL33-Re. The red arrow indicates the fragment size difference.

**Figure 3:**
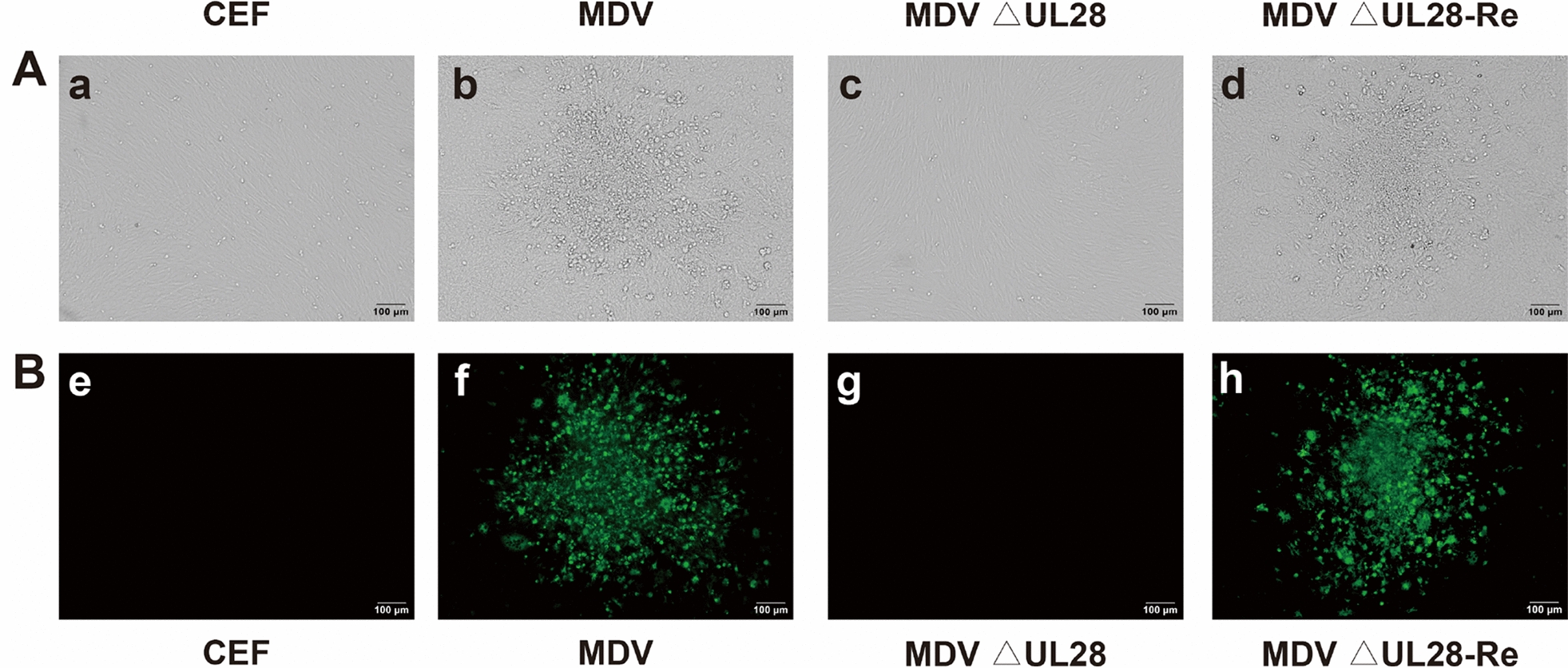
**Demonstration of the virus plaques in CEF transfected with DNAs of parental virus, MDV △UL28, MDV △UL28-Re.** **A** Plaque detection by regular light. Letter a represents un-transfected cells; Letter b represents DNA of parental virus transfected cells; Letter c represents MDV △UL28 transfected cells; Letter d represents MDV △UL28-Re transfected cells; **B** IFA analysis of MDV plaques in CEF. Images of e, f, g, h represent the same cell as images a, b, c, d, but stained with MDV specific gB monoclonal antibody and examined by fluorescent microscopy.

To study the role of UL28 and UL33 in MDV DNA packaging and replication, RecE/T-mediated mutagenesis was used to construct gene deletion mutant viruses, MDV ΔUL28 and MDV ΔUL33, in which the entire coding sequence of the UL28 or UL33 gene was deleted (Figure 2A). In detail, the KanR-I-SceI (kanamycin–I-SceI) cassette was amplified from pEPkan-S by specific primers with homologous sequences for downstream recombination events. After that, the gene sequences in the MDV genome were replaced with KanR-I-SceI cassette. In the next step, I-SceI expression was induced by arabinose to cleave the MDV-KanR, for the deletion of KanR followed by homologous recombination to generate MDV ΔUL28 and MDV ΔUL33. Second, revertant mutants (MDV UL28-Re and MDV ΔUL33-Re) were generated in which the gene sequence was completely restored in the deletion mutant. For the validation of the genomic changes introduced in the deletion and revertant viruses, the *UL28* or *UL33* gene was PCR amplified using the MDV-specific *Meq* gene as an internal control. As shown in Figures 2B and C, the *Meq* gene could be amplified in all of the tested constructs since it was not affected in the recombination process. The *UL28* or *UL33* gene was amplified in parental virus, revertant mutants, but not in MDV ΔUL28 and MDV ΔUL33. To further confirm that there were no major rearrangements in the BAC clones, parental, gene deletion and revertant viruses were subjected to restriction fragment length polymorphism (RFLP) analysis. The EcoRV and NdeI restriction fragment patterns were consistent with the predicted size changes in RFLP analysis (Figures 3D, E). These results confirmed the successful generation of the *UL28* and *UL33* deletion and revertant recombinant virus constructs.

### Characterization of MDV ΔUL28, MDV ΔUL33 and revertant viruses

**Figure 4:**
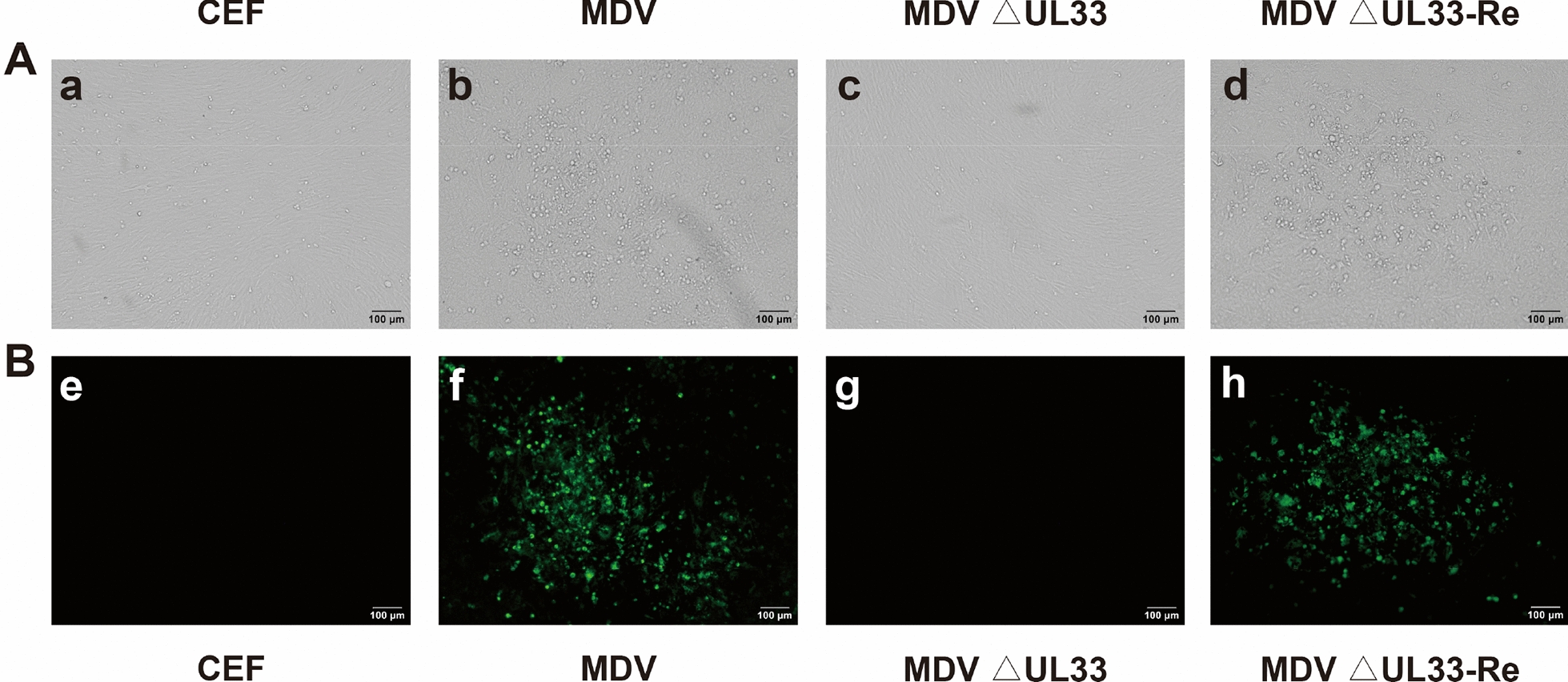
**Demonstration of the virus plaques in CEF transfected with DNAs of parental virus, MDV △UL33, MDV △UL33-Re.** **A** Plaque detection by regular light. Letter a represents un-transfected cells; letter b represents DNA of parental virus transfected cells; letter c represents DNA of MDV △UL33 transfected cells; letter d represents DNA of MDV △UL33-Re transfected cells; **B** IFA analysis of MDV plaques in CEF. Images of e, f, g, h represent the same cell as images a, b, c, d, but stained with MDV specific gB monoclonal antibody and examined by fluorescent microscopy.

For reconstitution of the recombinant viruses, 500 ng of BAC-viral DNA was transfected into CEF cells in 60-mm dish following calcium phosphate transfection procedure as previously described [23]. As expected, MDV-specific plaques became visible 3 days post-transfection of parental BAC-viral DNA, while no virus plaques were observed after transfection of the MDV ΔUL28 (Figure 3A) and MDV ΔUL33 (Figure 4A) BAC-viral DNA. The specificity of virus plaques was further confirmed by IFA assay using MDV specific anti-gB monoclonal antibody. As indicated in Figures 3B and 4B, the specific reactivity was observed in CEF transfected with DNA of parental virus, while no signals were detected in the MDV ΔUL28 and MDV ΔUL33 DNAs transfected CEF cells, as well as uninfected negative control CEF cells. In contrast, DNA transfection of MDV ΔUL28-Re and MDV ΔUL33-Re revertant constructs where *UL28* or *UL33* genes were restored, created plaques, similar to the parental virus. In order to rule out the possibility of low titers of virus after initial transfection, transfected cells with each mutant construct were passaged with fresh uninfected CEF cells. No MDV plaques or evidence of virus growth was directly observed or detected by IFA after inoculation of the MDV ΔUL28 transfected CEF cells, whereas MDV-specific plaques were obvious after inoculation with the parental or revertant mutants transfected CEF cells. All of the transfection and co-inoculating experiments were repeated more than three times to confirm the original results.

### Rescue of deletion mutant viruses after co-transfection of UL28 or UL33 DNA

**Figure 5:**
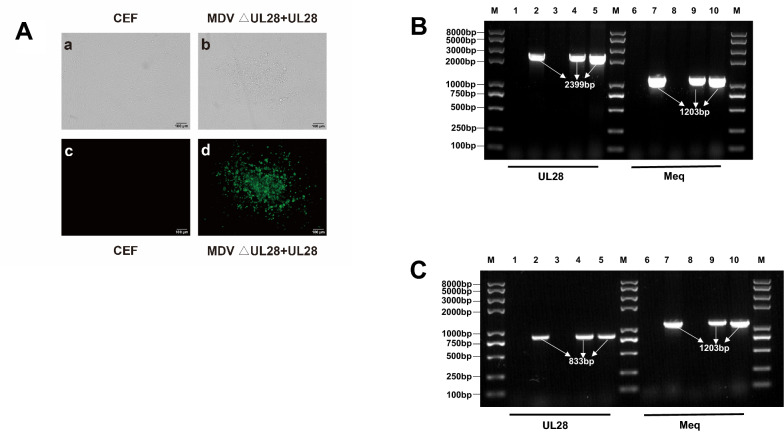
**Identification of the MDV ΔUL28 and UL28 gene co-transfected viruses.** Cellular DNA and RNA were extracted from the uninfected, recombinant viruses of the parental virus, MDV △UL28, MDV △UL28-Re or MDV △UL28 and UL28 co-transfection produced recombinant viruses infected CEF cells, respectively. **A** The *UL28* and *meq* gene were PCR amplified using the cellular DNA as template. lane 1and 6 represents uninfected cell; lane 2 and 7 represents parental virus; lane 3 and 8 represents MDV △UL28; lane 4 and 9 represents parental virus; lane 5 and 10 represents MDV △UL28 and UL28 co-transfection; **B** The *UL28* and *meq* gene were RT-PCR amplified using the cellular RNA as template. lane 1 and 6 represents uninfected cell; lane 2 and 7 represents parental virus; lane 3 and 8 represents MDV △UL28; lane 4 and 9 represents parental virus; lane 5 and 10 represents MDV △UL28 and UL28 co-transfection; **C** Virus plaque examination of MDV △UL28 and UL28 co-transfection in CEF cells. No plaque was examined and observed in uninfected CEF cells in a (regular light) and b (fluorescent light); MDV specific plaques were examined and observed in the MDV △UL28 and UL28 co-transfected CEF cells in c (regular light) and d (fluorescent light).

Results from the transfection of different constructs clearly demonstrated that UL28 and UL33 are necessary for MDV replication. To further verify whether expression of *UL28* or *UL33* indeed can restore the growth ability of the gene deletion mutants in cultured cells, purified *UL28* or *UL33* gene fragments amplified by PCR were co-transfected with MDV △UL28 or MDV △UL33 BAC-DNA into CEF cells. At day 7 after transfection, no visible MDV-specific plaques were detected in the MDV △UL33 and *UL33* gene co-transfected group. However, MDV-specific plaques were observed in the MDV △UL28 and *UL28* gene co-transfected groups (Figure 5A, upper), which were further confirmed by IFA test (Figure 5A, lower). PCR analysis on the genomic DNA showed that the *UL28* gene could be amplified from the MDV △UL28 and *UL28* co-transfected group (Figure 5B). To test whether the *UL28* gene indeed was expressed, total RNA was extracted and RT-PCR analysis indicated that the *UL28* specific transcript could be detected in the co-transfected group (Figure 5C). These results further confirmed that the transient expression of the *UL28* gene into MDV △UL28 also restored the function of viral replication.

### Characterization of the recombinant viruses

**Figure 6:**
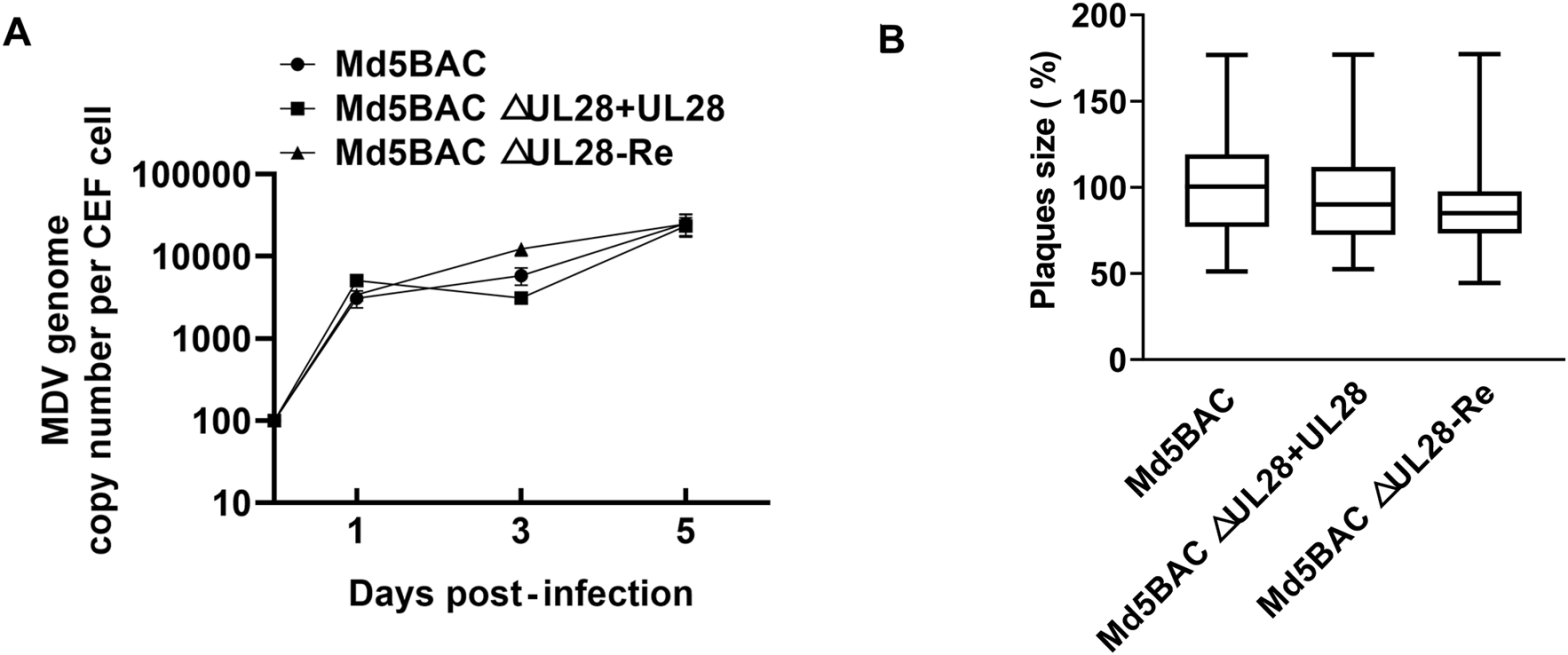
**Characterization of the parental virus, MDV △UL28-Re, MDV △UL28 and UL28 co-transfection resulted recombinant viruses.** **A** Growth kinetics of recombinant viruses was examined by inoculation with 100 PFUs of the indicated virus into CEF cells, respectively. The infected CEF cells were trypsinized and seeded on fresh cells on days 0, 1, 2, 3, 4, 5, and 6 post-inoculation. MDV-specific plaques were counted at 7 days post-inoculation on fresh CEF for virus titers calculation. The experiment was performed in duplicate, and virus titer is indicated as PFUs for each 35-mm dish. Results represent mean values with error bars showing the standard error of the mean; **B** CEF cells were infected with 100 PFU per 60 mm dishes of parental, revertant or co-transfected mutant viruses and used to determine the plaque areas. Seven days post-infection, the infected cells were stained with MDV specific anti-gB monoclonal antibody and examined under fluorescence microscope. The virus plaque areas were measured by ImageJ software. Results represent mean values with error bars showing the standard error of the mean.

We then asked whether the recombinant viruses altered replication properties due to mutagenesis. After amplification and titration of the recombinant viruses, the growth of MDV mutants was determined by measurement of both growth curve in vitro and plaque area. As indicated in Figure 6, the MDV △UL28-Re and *UL28* PCR product co-transfected viruses had the similar growth property with parental virus. These results indicated that UL28 re-expression completely restored the replication ability of mutant viruses.

### The UL28 deficiency results in immature virions

**Figure 7:**
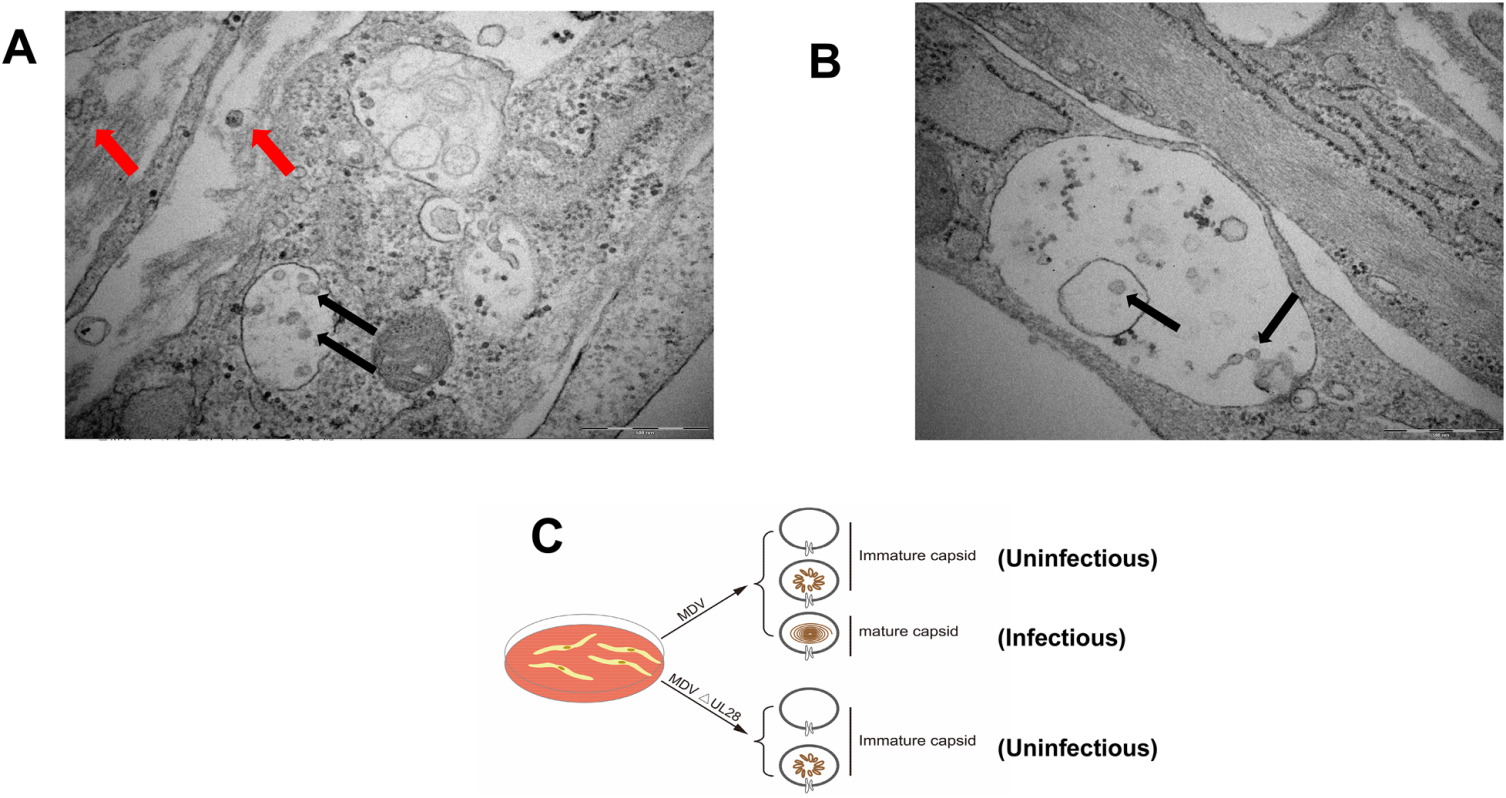
**Electron microscopy examination of parental virus-infected or DNA of MDV △UL28-transfected CEF cells.** **A** CEF cells were infected with parental virus or transfected with DNA of MDV △UL28, and fixed at 3 days post-infection/transfection. The electron microscopic examination indicated that mature and immature virions were formed. The mature virions were marked with a red arrow, while immature virions were marked with a black arrow; **B** CEF cells were transfected with DNA of MDV △UL28, and fixed at 3 days post-transfection. The electron microscopic examination revealed that immature capsids were formed and marked with a black arrow. The scale bar is 500 nm.

Since both the UL28 revertant and *UL28* co-transfection were able to form virus plaques on CEF after transfection of the mutant DNA, we questioned whether capsids were produced during UL28-null mutant transfection. For comparison, we first infected CEF cells with the parental virus. At 3 days post-infection, the infected cells were fixed and examined by transmission electron microscopy. As shown in Figure 7A, both mature and immature capsids could be observed, suggesting natural morphogenesis in the parental virus infected CEF cells. In the MDV △UL28 mutant transfected CEF cells, however, only naked procapsids were observed when transfected CEF cells were examined by transmission electron microscopy at 3 days post-transfection (Figure 7B). From the results above, we concluded that UL28-null virus produces immature capsid but loses infectivity because of the deficiency of DNA packaging (Figure 7C).

### MDV and HSV-1 encoded UL28 shares conserved tertiary structure

**Figure 8:**
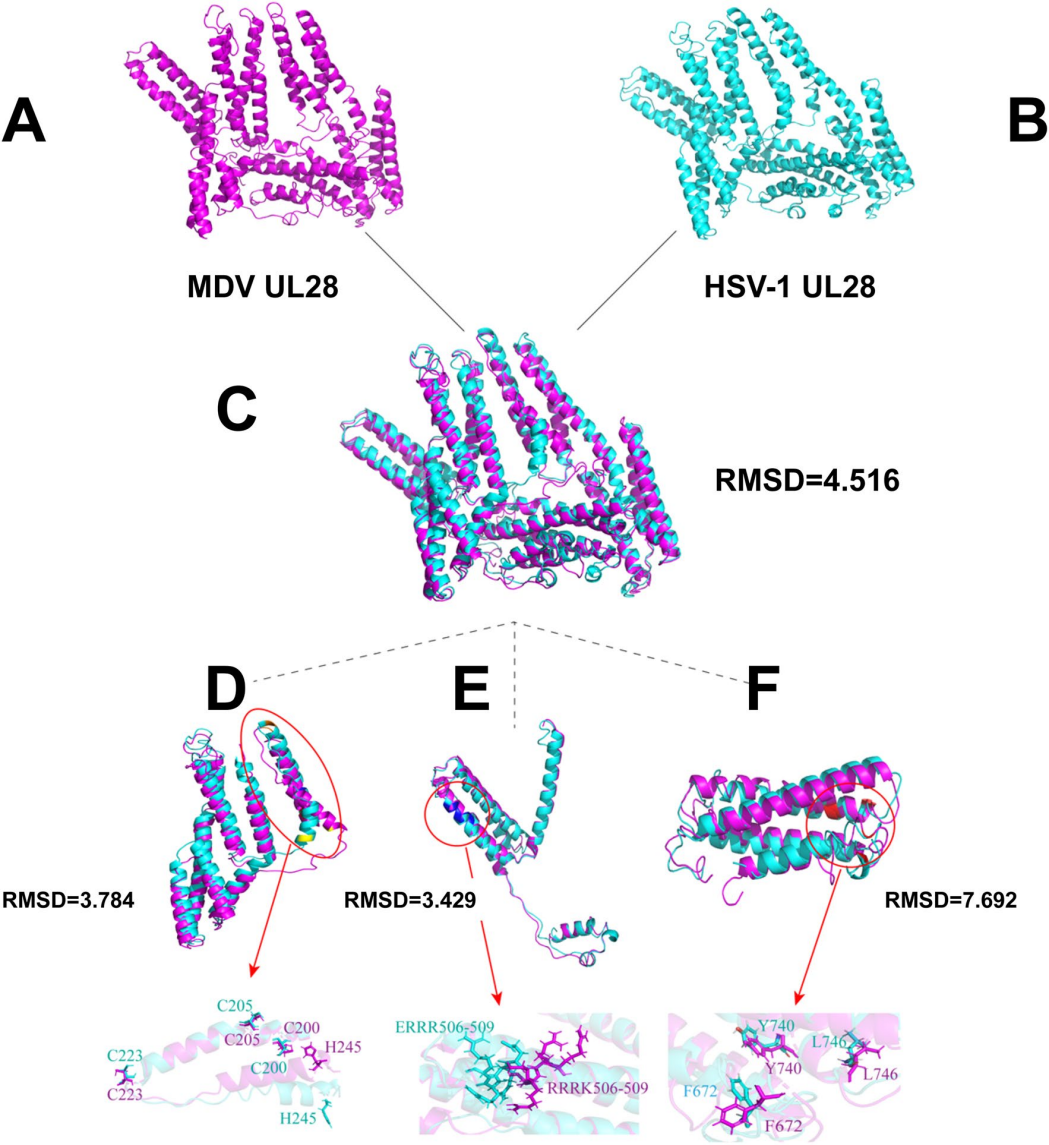
**Tertiary structure analysis of MDV and HSV-1 encoded UL28.** **A** Predicted tertiary structure of MDV encoded UL28; **B** Predicted tertiary structure of HSV-1 encoded UL28; **C** Alignment of the predicted tertiary structure of MDV and HSV-1 encoded UL28; **D** Overlapping of predicted tertiary structure of the putative zinc-finger motif; **E** Overlapping of predicted tertiary structure of the 506-RRQR-509 motif; **F** Overlapping of predicted tertiary structure of the C-terminal motif.

Both of MDV and HSV-1 encoded UL28 proteins shared highly conserved amino acid sequences, and induced virus replication deficiency once mutated. We questioned whether MDV and HSV-1 encoded UL28 proteins also have closed tertiary structure to execute similar biological functions. The tertiary structural model of MDV and HSV-1 encoded UL28 proteins were predicted by the I-TASSER server and analyzed by Pymol (Figures 8A and B). The UL28 tertiary structure of both viruses was similar in basic structure of alpha-helix (RMSD = 4.516) (Figure 8D). For the specific conserved motif analysis, we divided the structure of the UL28 protein into four parts. In the putative zinc-finger motif (C-X2-C-X22-C-X–H), the conformation of C200, C205 and C223 amino acids were completely matched, while H245 was different in MDV and HSV-1 encoded UL28 proteins (Figure 8E). The conformation of the 506-RRQR-509 motif was different (Figure 8E). In the C-terminal motif, most of amino acid sequences were not consistent (Figure 8F). The alignment of predicted tertiary structure of UL28 in MDV and HSV-1 further confirmed that the similar function in DNA cleavage and packaging of these two herpesviruses, but may not be in nuclear location and translocalization.

## Discussion

During herpesvirus replication, an assembled icosahedral capsid was initially formed, and then viral genomic DNA was packaged into through the entrance and exit pore of portal vertex, which is driven by terminase complex [12]. The UL28 and UL33 proteins are key components of the terminase machinery. Once deficient in the components of this machinery, the viral genomic DNA cannot be packaged into and no infectious viruses can be formed [24]. In the present study, we have predicted and conducted experiments to identify the role of the UL28 and UL33 in MDV replication in vitro. Deletion of either *UL28* or *UL33* gene resulted in no infectious virion formation in cultured cells (Figure 4). Furthermore, the immature capsids were observed after MDV △UL28 BAC-DNA transfection (Figure 7). These findings indicated that the UL28 and UL33 are essential for virus replication.

The HSV-1 *UL28* gene encoding 785 aa protein is essential for replication as partial *UL28* deletion mutants were unable to form viral plaques or produce infectious virus in cultured cells without the UL28 expression [25]. Confirmed by transmission electron microscopy assay, the *UL28* deletion mutant was defective in cleavage and encapsidation of viral genomic DNA [26]. As the smallest subunit of terminase, the HSV-1 UL33-null mutant showed disability of replication, which may be determined by specific motif of the UL33 [27]. Previous study also demonstrated that the interaction of the UL28 and UL15 protein for the DNA cleavage and packaging in B capsid might be regulated by highly conserved regions of the UL33 [22, 25, 28]. When expressed individually, UL15 and UL28 proteins localized in the nuclear and cytoplasm, respectively. However, co-expression of the two proteins resulted in the translocation of UL28 to the nucleus [29]. Similarly, in PRV replication, the UL28 also interacted with the proteins UL6, UL15, UL32 and UL33. Interestingly, the UL6, UL15 and UL32 were located in the nucleus, while the UL28 and UL33 remained in cytoplasmic [30]. In addition, studies have also shown that at least two separate regions of the UL28 could interact with the UL15. The trans-complementing of one part of region could rescue phenotype of DNA packaging and virus replication [31], suggesting the specific interaction between UL28 and other proteins may exert different functions in DNA cleavage and packaging, which may be controlled by specific and different motifs of the UL28 polypeptide chain. In our study, complete abolishing of MDV replication seen in UL28 or UL33-null mutants might be through disturbing DNA cleavage and packaging. However, the specific role of each conserved region in UL28 and UL33 need to be further investigated by mutational analysis of these genes.

In Bovine herpesvirus 1 (BoHV-1), deletion of the *UL28* gene neither compromised procapsid formation, nor abolished virus DNA replication. However, no infectious virions were assembled, because of the defect in the process of cleavage of the newly synthesized DNA and DNA packaging. These results demonstrated that the BoHV-1 UL28 is essential for viral replication and is necessary for the formation of mature capsid [32]. In contrast, procapsids were formed in CEF transfected with the UL28-deficient MDV mutant in our studies examining the functions of MDV UL28 (Figure 7). No viral genomic DNA and viral transcripts were detected in the MDV △UL28-transfected CEF cells, indicating that MDV genomic DNA replication in cultured cells was completely impaired (Figure 5). It has been demonstrated that UL28 is essential for HSV-1 replication. The predicted tertiary structure of the UL28 was similar in MDV and HSV-1 may be a further proof of similar functions in virus replication, although the real crystal structures need to be compared.

Although several human herpesviruses cause fatal diseases, there are few effective drugs for clinical therapy. For example, KHSV and EBV infections are responsible for 5% of tumorigenesis in humans [33], but effective antiviral drugs are still not available for clinical use. Capsid assembly and DNA packaging are potential pharmaceutic target to inhibit herpesvirus replication. As the terminase components [13], the UL28 and UL33 play an important role in herpesvirus replication, these could be explored as novel therapeutic targets. We found that MDV UL28-null mutant completely lost the ability of replication, which is consistent with HSV-1 UL28 deletion mutants (Figure 3). Furthermore, the predicted tertiary structure of MDV and HSV-1 encoded UL28 shared conserved putative zinc-finger motif, which is critical for viral genome DNA cleavage and packaging (Figure 8). These results suggested that the zinc-finger motif in UL28 could be as a potential target for drug development. Lack of suitable animal models of diseases are often underlined as a limitation for drug development. As highlighted previously, MDV infections and associated lymphomagenesis in its natural chicken host could be useful as an excellent model for the UL28 and UL33-targeted drug development in the future [3].

In summary, we constructed MDV *UL28* and *UL33*-deficient and revertant mutants to examine the functions of both genes. Using a transfection infection model in CEF cells, we proved that the *UL28* and *UL33*-deficient mutant could not be packaged, compared to the parental and revertant viruses. Furthermore, we showed that the packaging and replication defect of the gene-deletion mutant, could be restored by transit expression of the corresponding genes. These results confirmed that UL28 and UL33 are indispensable in MDV packaging and replication.

## Data Availability

All data generated or analyzed during this study are included in this submitted manuscript.

## Abbreviations

HSV-1: Herpes simplex virus 1
MDV: Marek’s disease virus
CEF: Chicken embryo fibroblast
dsDNA: Double-stranded DNA
HVT: Herpesvirus of turkeys
UL: Unique long
US: Unique short
TRL/IRL: Terminal and inverted repeat long
TRS/IRS: Terminal and inverted repeat short
ATP: Adenosine triphosphate
PCR: Polymerase chain reaction
SPF: Specific pathogen free
DMEM: Dulbecco’s modified essential medium
FBS: Fetal bovine serum
qPCR: Real-time quantitative PCR detecting system
LB: Luria–Bertani
IFA: Indirect Immunofluorescence
PBS: Phosphate-buffered saline
FITC: Fluorescein isothiocyanate
ORF: Open Reading Frame
PFUs: Plaque-forming units
RFLP: Restriction fragment length polymorphism
PRV: Pseudorabies virus
BoHV-1: Bovine herpesvirus 1
KSHV: Kaposi’s Sarcoma Associated Herpesvirus
EBV: Epstein-Barr virus
